# Supplementary material for: Gut microbiota of obese and diabetic Thai subjects and interplay with dietary habits and blood profiles
Source: PeerJ. 2020 Aug 3;8:e9622. doi: 10.7717/peerj.9622 (PMC7409811; doi:10.7717/peerj.9622)
Supplement: Supplemental Information 1 — The statistical significance of differences in the mean ranks among groups was determined using Kruskal-Wallis rank sum test with Benjamini–Hochberg procedure (p value adjusted < 0.05). [file peerj-08-9622-s001.docx]

**Table S1:**

**The frequency of dietary consumption of subjects in BMI and T2DM groups.**

| **Food item** | **Mean of consumption frequency ^a^ (times per week)** | | | | | |
| --- | --- | --- | --- | --- | --- | --- |
|  | **Lean**  **(n = 8)** | **OV**  **(n = 8)** | **OB**  **(n = 7)** | **T2DM**  **(n = 7)** | ***p*-value^b^** | **BH^c^** |
| Pork | 4.12 ± 1.13 | 4.12 ± 0.99 | 4.86 ± 1.21 | 3.43 ± 0.10 | 0.171 | 0.228 |
| Chicken | 3.38 ± 0.74 | 3.00 ± 0.76 | 4.29 ± 1.25 | 2.29 ± 0.49 | 0.005 | 0.039 |
| Fish | 4.25 ± 1.04 | 4.25 ± 1.16 | 4.14 ± 1.35 | 5.43 ± 0.79 | 0.121 | 0.228 |
| Beef | 2.38 ± 0.92 | 1.88 ± 1.25 | 2.71 ± 1.60 | 1.29 ± 0.49 | 0.095 | 0.228 |
| Egg | 4.00 ± 0.93 | 4.00 ± 0.76 | 4.57 ± 1.13 | 3.43 ± 0.54 | 0.152 | 0.228 |
| Dairy products | 2.00 ± 1.85 | 2.75 ± 1.75 | 3.29 ± 1.60 | 1.43 ± 0.79 | 0.110 | 0.228 |
| Pea/nut/bean | 2.62 ± 1.06 | 2.38 ± 0.74 | 3.43 ± 1.27 | 2.43 ± 0.98 | 0.302 | 0.345 |
| Yogurts/Cheese/fermented milk | 2.50 ± 1.60 | 2.25 ±1.28 | 3.14 ± 1.21 | 3.43 ± 1.27 | 0.435 | 0.435 |
| Sticky rice | 5.62 ± 0.74 | 5.00 ± 1.51 | 5.00 ± 1.29 | 3.86 ± 2.12 | 0.304 | 0.773 |
| Steamed rice | 3.88 ± 1.55 | 3.75 ± 1.83 | 4.43 ± 1.51 | 4.86 ± 1.35 | 0.591 | 0.773 |
| Brown rice | 1.38 ± 0.74 | 1.75 ± 1.49 | 1.71 ± 1.11 | 2.29 ± 1.89 | 0.765 | 0.773 |
| Mixed rice | 2.00 ± 1.77 | 2.75 ± 1.98 | 1.71 ± 1.25 | 2.14 ± 1.86 | 0.773 | 0.773 |
| Rice vermicelli | 3.25 ± 0.71 | 3.00 ± 1.07 | 2.14 ± 1.21 | 1.86 ± 0.69 | 0.028 | 0.168 |
| Bread | 2.12 ± 1.13 | 2.75 ± 1.49 | 2.43 ± 1.62 | 1.86 ± 1.07 | 0.604 | 0.773 |
| Grain | 2.25 ± 0.71 | 2.12 ± 1.13 | 3.00 ± 1.41 | 2.43 ± 1.27 | 0.631 | 0.631 |
| Green vegetable | 4.50 ± 1.60 | 5.38 ± 0.92 | 4.86 ± 1.57 | 5.86 ± 0.38 | 0.143 | 0.357 |
| Fermented fruits/vegetable | 3.62 ± 1.85 | 1.75 ± 0.71 | 3.29 ± 1.50 | 1.71 ± 0.76 | 0.038 | 0.189 |
| Specified vegetables | 4.62 ± 0.74 | 4.88 ± 1.25 | 4.00 ± 1.29 | 3.86 ± 1.77 | 0.419 | 0.523 |
| Fruit | 3.25 ± 1.49 | 4.25 ± 1.39 | 4.43 ± 1.40 | 3.86 ± 1.46 | 0.390 | 0.523 |
| Tea | 2.00 ± 1.93 | 2.00 ± 1.85 | 2.71 ± 1.89 | 2.29 ± 2.21 | 0.735 | 0.735 |
| Coffee | 4.12 ± 1.81 | 4.00 ± 2.51 | 4.57 ± 1.90 | 3.00 ± 2.52 | 0.669 | 0.735 |
| Carbonate soft drink | 2.00 ± 1.51 | 2.12 ± 1.81 | 2.43 ± 1.62 | 1.14 ± 0.38 | 0.370 | 0.724 |
| Juice | 3.12 ± 1.81 | 3.00 ± 1.51 | 3.29 ± 1.50 | 2.14 ± 1.68 | 0.483 | 0.724 |
| Energy drink | 2.38 ± 1.60 | 1.75 ± 1.75 | 3.00 ± 2.00 | 1.57 ± 1.51 | 0.202 | 0.607 |
| Alcoholic beverage | 3.50 ± 1.60 | 2.88 ± 1.55 | 2.71 ± 1.38 | 1.71 ± 1.89 | 0.073 | 0.435 |

^a^Mean ± SD

^b^Kruskal-Wallis rank sum test (*p*-value < 0.05)

^c^Benjamini–Hochberg method (*p*-value adjusted < 0.05)
