# Supplementary figures and images for: Gut microbiota of obese and diabetic Thai subjects and interplay with dietary habits and blood profiles
Source: PeerJ. 2020 Aug 3;8:e9622. doi: 10.7717/peerj.9622 (PMC7409811; doi:10.7717/peerj.9622)

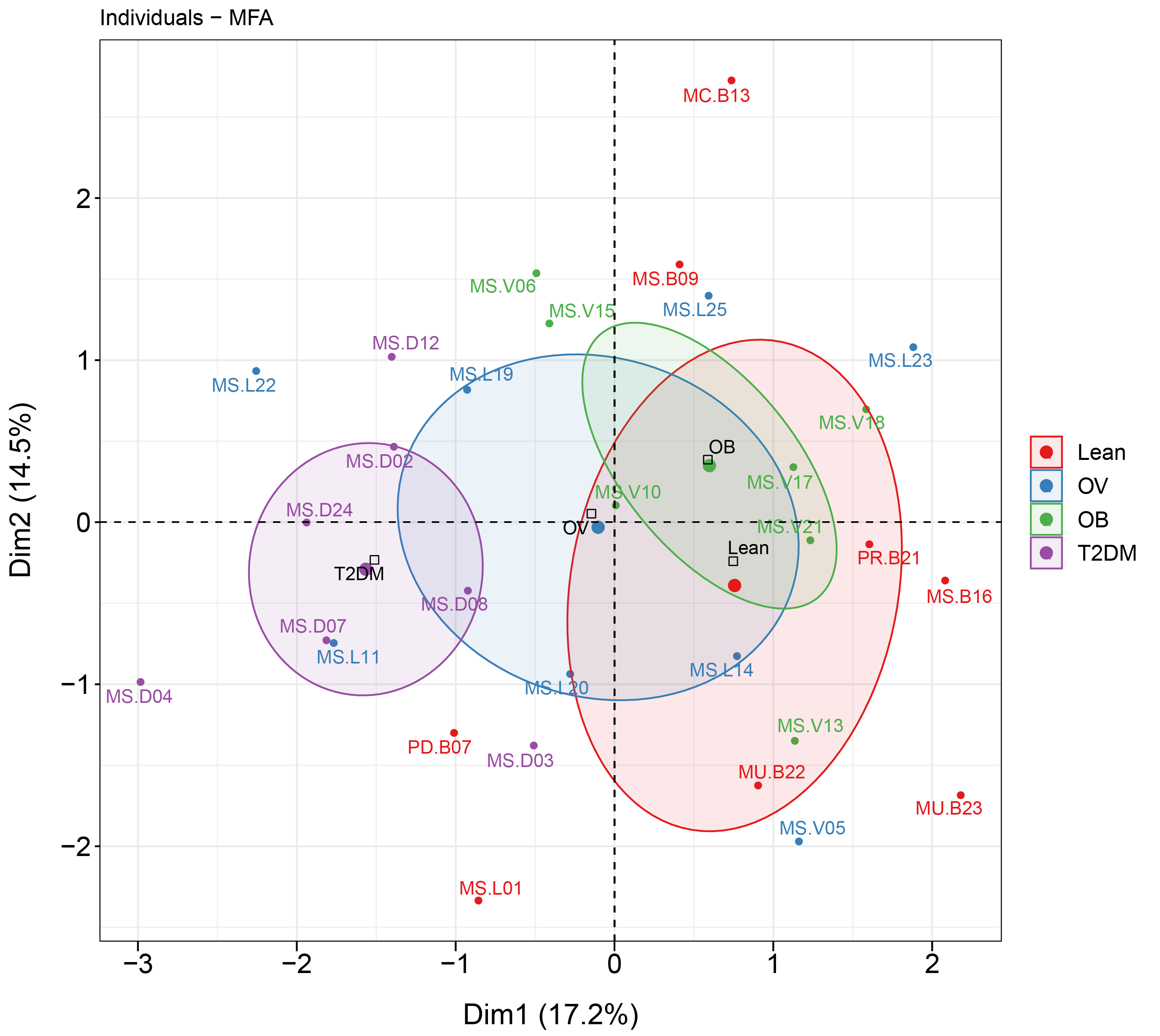

Supplement: Supplemental Information 4 — The factor map presents the frequency of food consumption profiles in different BMI groups and T2DM subjects based on MFA. The coordinates of the individuals are indicated by the 95% confidence ellipses including orange (Lean), blue (OV), green (OB), and purple (T2DM). [file peerj-08-9622-s004.png]

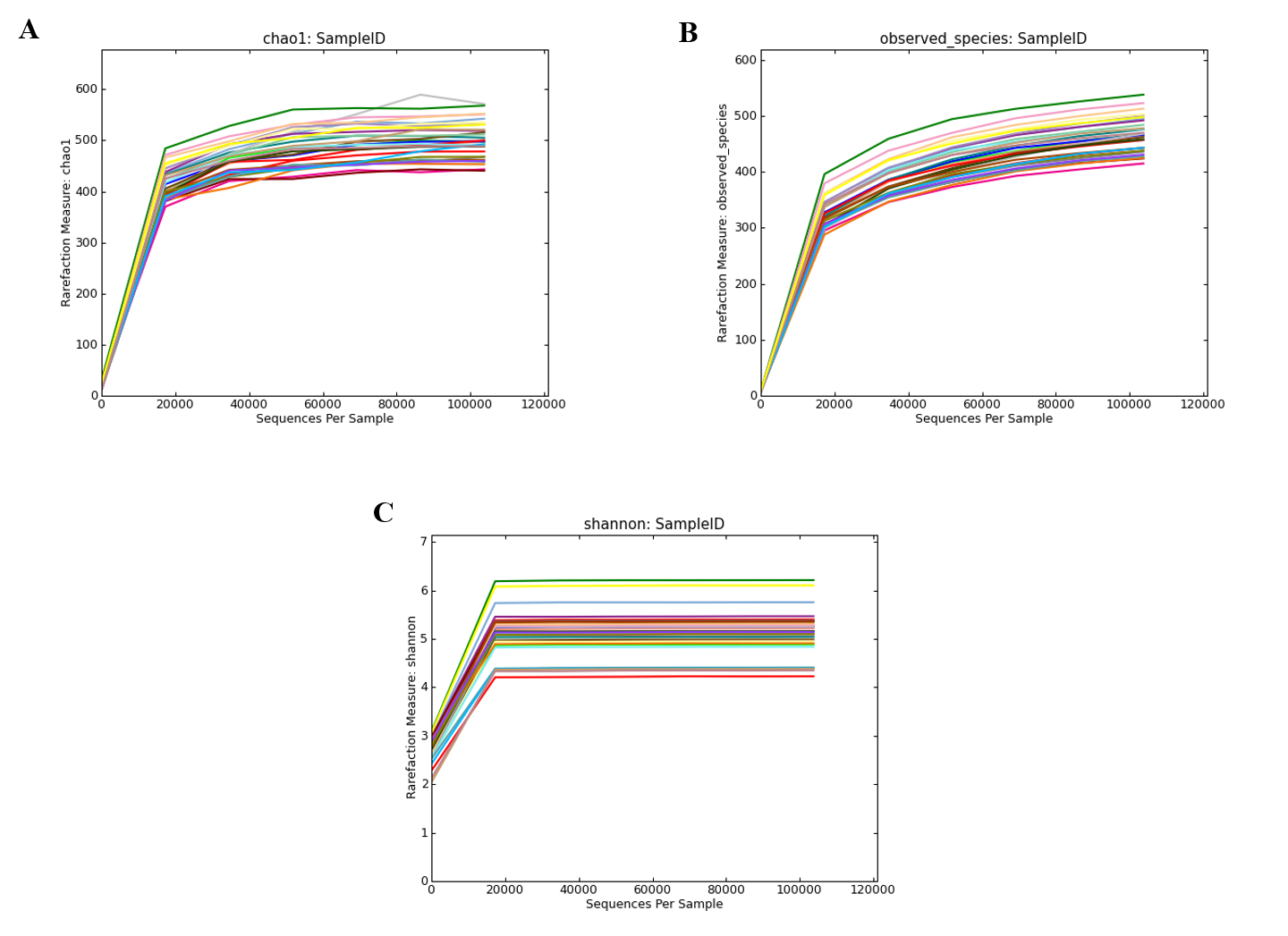

Supplement: Supplemental Information 5 — Sampling curves generated for each fecal sample show the relationship between a diversity index (vertical axis) and sequencing depth (horizontal axis). (A) Rarefaction curve based on Chao1. (B) Rarefaction curve based observed species. (C) Rarefaction curve based Shannon. Each curve is represented by a unique color. [file peerj-08-9622-s005.png]

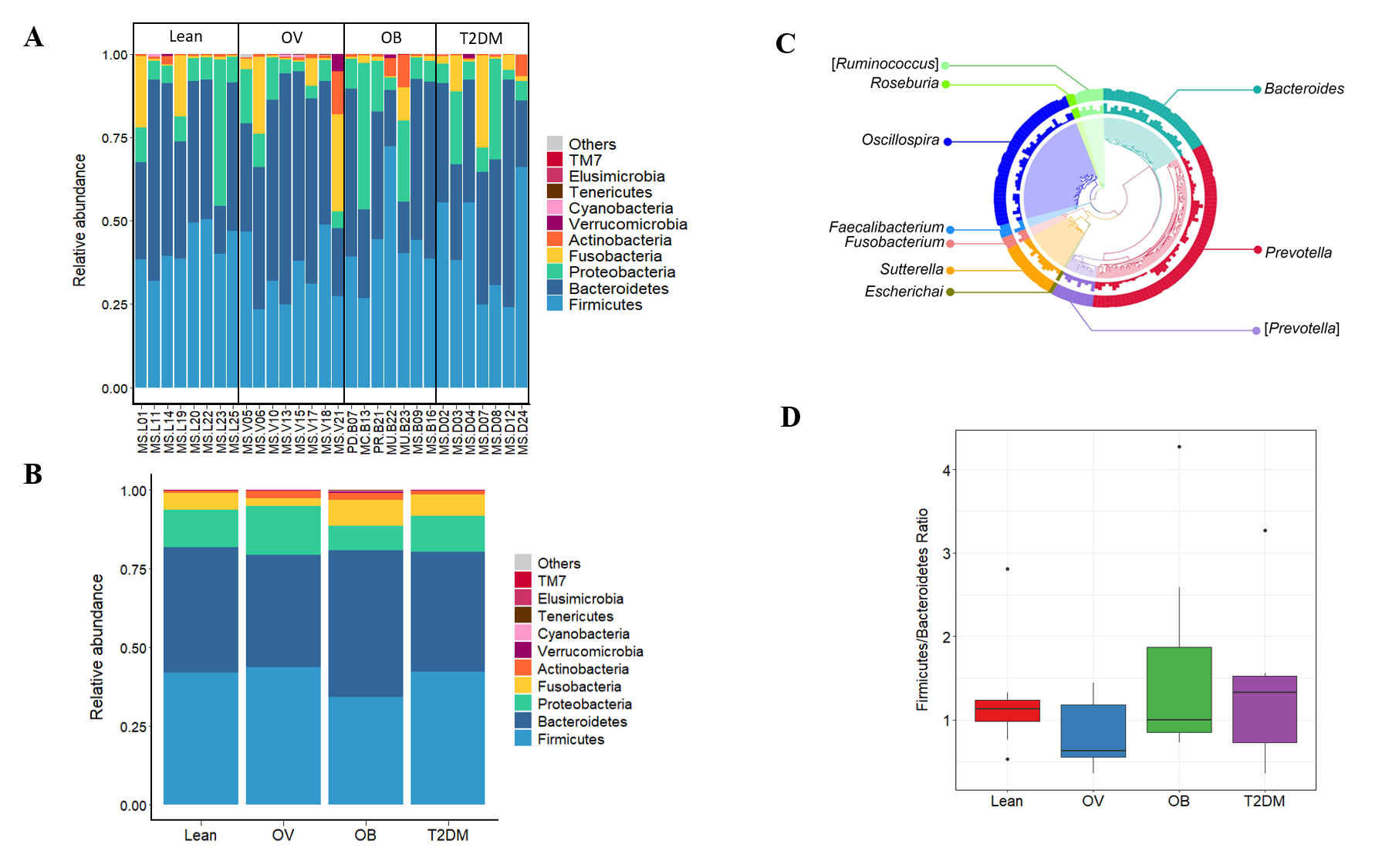

Supplement: Supplemental Information 6 — (A) Relative abundance of fecal gut microbiome at phylum level presented in each subject. (B) Relative abundance of fecal gut microbiome at phylum level presented in each group. (C) OTU annotation tree of bacterial genera. (D) Boxplot of Firmicutes to Bacteroidetes ratio across four groups (asterisks indicate p < 0.05, Wilcoxon rank-sum test). [file peerj-08-9622-s006.png]

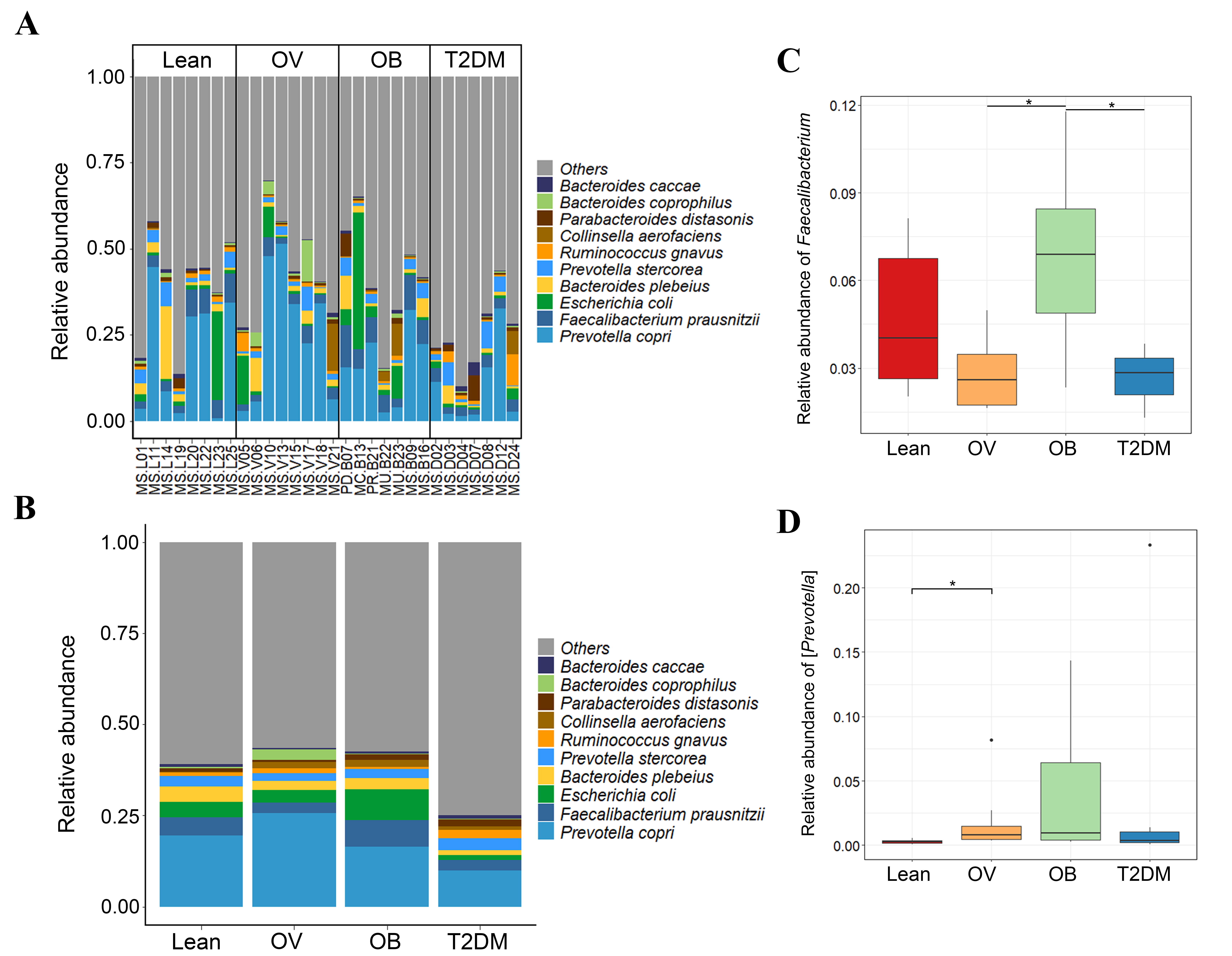

Supplement: Supplemental Information 7 — (A) Relative abundance of fecal gut microbiome at species level presented in each subject. (B) Relative abundance of fecal gut microbiome at species level presented in each group. (C) Boxplot of relative abundance of Faecalibacterium prausnitzii across four groups. (D) Boxplot of relative abundance of Prevotella copri across four groups. Asterisks indicate p < 0.05, Wilcoxon rank-sum test without Benjamini–Hochberg method. [file peerj-08-9622-s007.png]

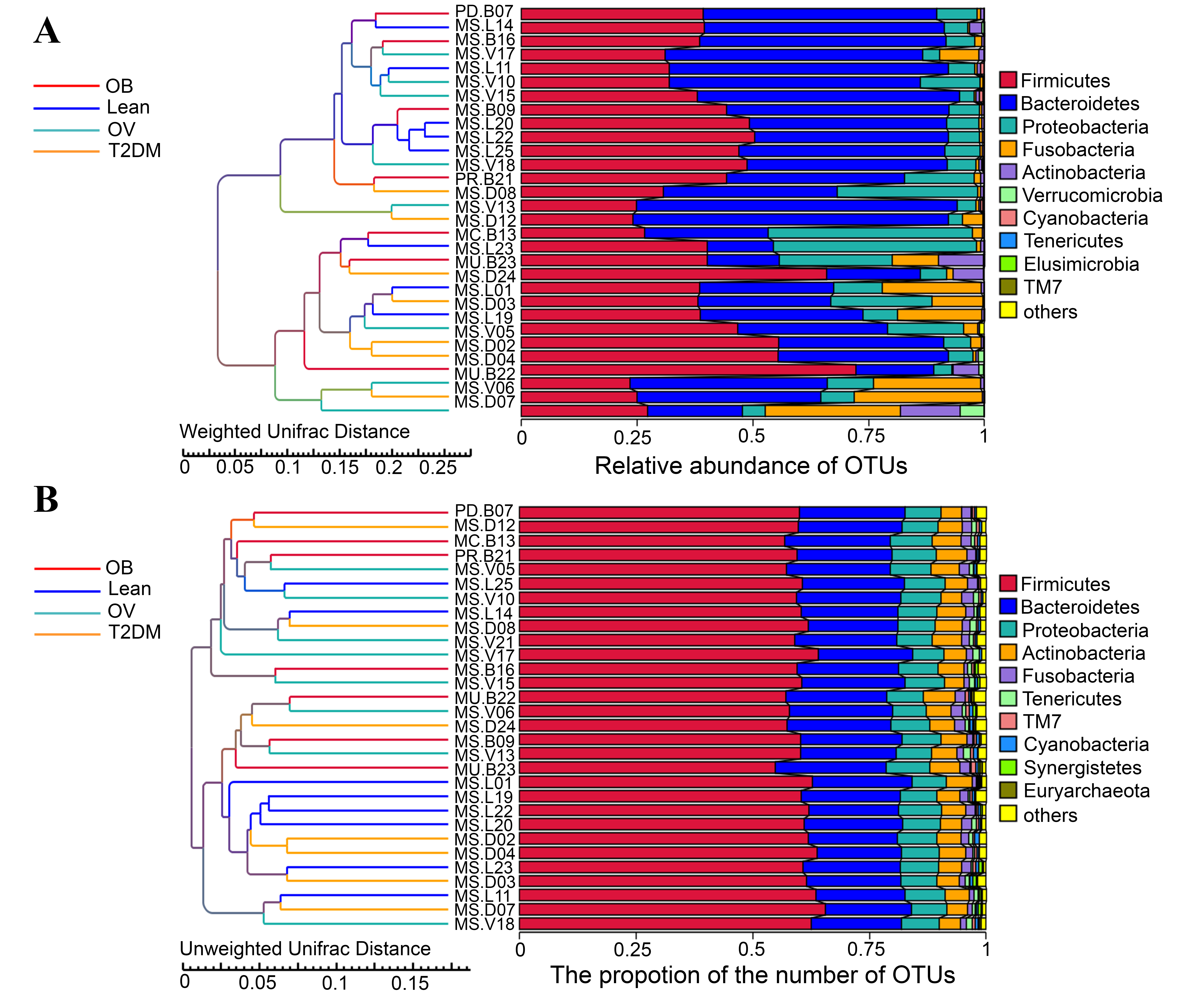

Supplement: Supplemental Information 8 — Differences of fecal bacterial community structure between BMI and T2DM groups evaluated by beta diversity analysis. (A) UPGMA cluster tree based on Weighted UniFrac distance. (B) UPGMA cluster tree based on Unweighted UniFrac distance (below). Subjects from L, OV, OB, and T2DM groups are presented in blue, green, red, and yellow color, respectively. [file peerj-08-9622-s008.png]

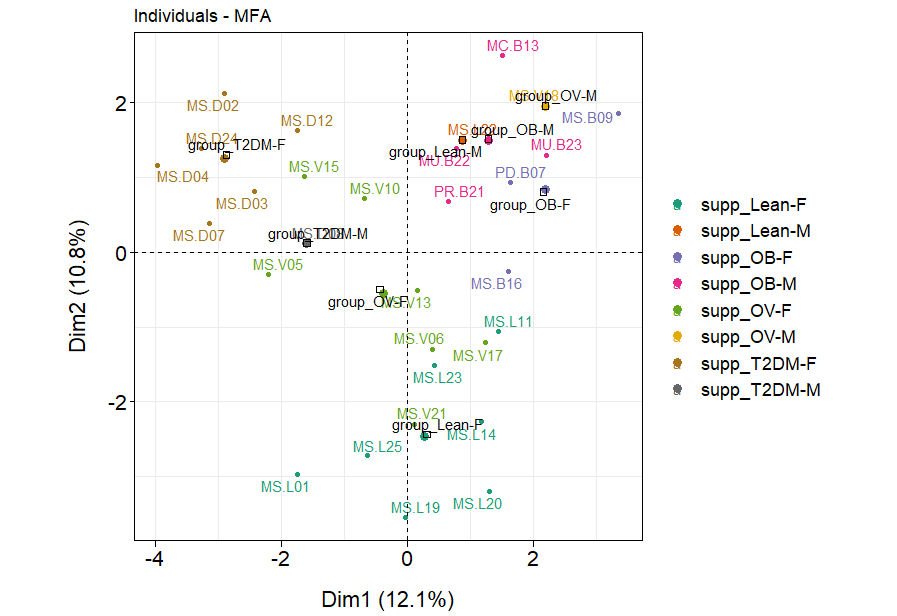

Supplement: Supplemental Information 9 — The factor map presents the integration of gender, age, blood profiles (HDL and FBS), dietary habits, and fecal gut microbiota (at genus level) of subjects in different BMI groups and T2DM group based on the MFA. F: a female individual, M: a male individual. [file peerj-08-9622-s009.png]

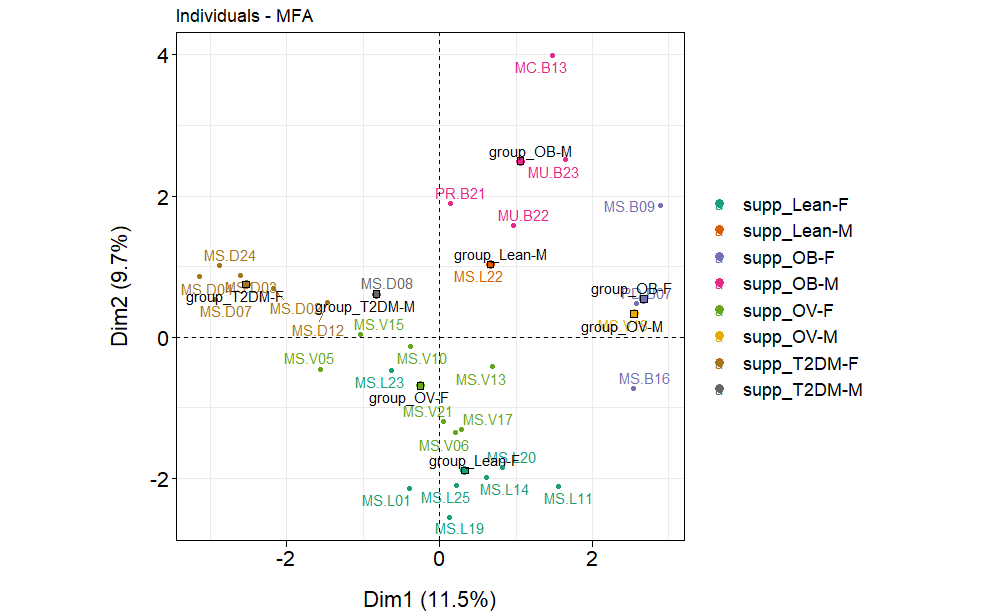

Supplement: Supplemental Information 10 — The factor map presents the integration of gender, age, all blood profiles, dietary habits, and fecal gut microbiota (at genus level) of subjects in different BMI groups and T2DM group based on the MFA. F: a female individual, M: a male individual. [file peerj-08-9622-s010.png]

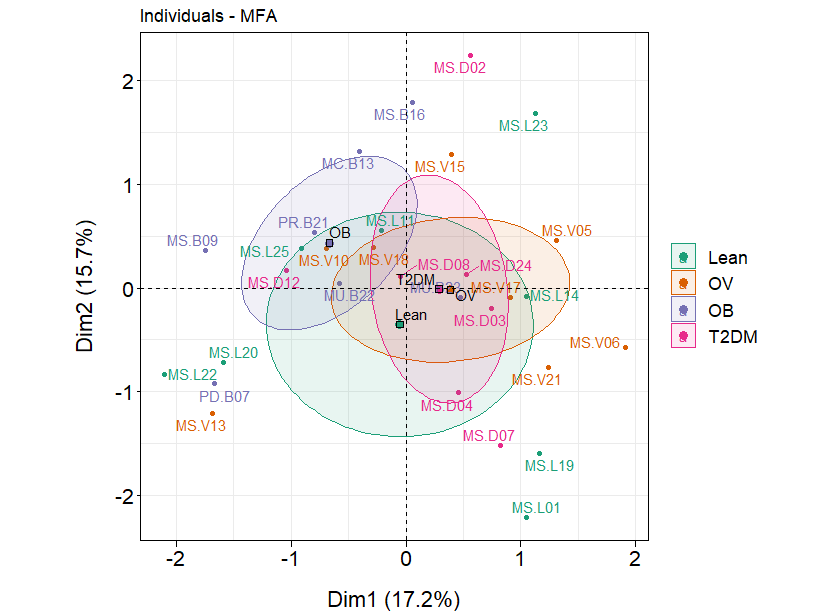

Supplement: Supplemental Information 11 — The factor map presents the integration of all blood profiles and fecal gut microbiota (at genus level) of subjects in different BMI groups and T2DM group based on the MFA. The coordinates of the individuals are indicated by the 95% confidence ellipses including green (L), orange (OV), purple (OB), and pink (T2DM). [file peerj-08-9622-s011.png]
