# Supplementary material for: Gut microbiota of obese and diabetic Thai subjects and interplay with dietary habits and blood profiles
Source: PeerJ. 2020 Aug 3;8:e9622. doi: 10.7717/peerj.9622 (PMC7409811; doi:10.7717/peerj.9622)
Supplement: Supplemental Information 12 [file peerj-08-9622-s012.pdf]

Participant characteristics

No.	Questions/Answers	Variable name	Results
1	Code of volunteer □□ – □ – □□	Code	
2	Birth date dd/mm/year □□ – □□ – □□□□	Birth date	
3	Age □□ year	Age	
4	Gender □ 1) Male □ 2) Female	Gender	
5	Blood pressure (Systolic BP) □□□ / □□□ mmHg	SBP	
6	Blood pressure (Diastolic BP) □□□ / □□□ mmHg	DBP	
7	Weight □□□ . □□ kg	Weight	
8	Height □□□ . □□ cm	Height	

Food frequency questionnaire (FFQ)

No.	Questions	Answer	Every day	5-6 days	3-4 days	1-2 days	Less than once a week	Never	Variable name	Type	Results
		Frequency per week									
		Score	6	5	4	3	2	1			
1	Pork								OQ-1	Ordinal	
2	Chicken								OQ-2	Ordinal	
3	Fish								OQ-3	Ordinal	
4	Beef								OQ-4	Ordinal	
5	Egg								OQ-5	Ordinal	
6	Dairy products								OQ-6	Ordinal	
7	Pea/nut/bean								OQ-7	Ordinal	
8	Yogurts/Cheese/fermented milk								OQ-8	Ordinal	
9	Sticky rice								OQ-9	Ordinal	
10	Steamed rice								OQ-10	Ordinal	
11	Brown rice								OQ-11	Ordinal	
12	Mixed rice								OQ-12	Ordinal	
13	Rice vermicelli								OQ-13	Ordinal	
14	Bread								OQ-14	Ordinal	
15	Grain								OQ-15	Ordinal	
16	Green vegetable								OQ-16	Ordinal	
17	Fermented fruits/vegetable								OQ-17	Ordinal	
18	Specified vegetables								OQ-18	Ordinal	
19	Fruit								OQ-19	Ordinal	
20	Tea								OQ-20	Ordinal	
21	Coffee								OQ-21	Ordinal	
22	Carbonate soft drink								OQ-22	Ordinal	
23	Juice								OQ-23	Ordinal	
24	Energy drink								OQ-24	Ordinal	
25	Alcoholic beverage								OQ-25	Ordinal	
